# *Treponema pallidum* protein Tp0136 promotes angiogenesis to facilitate the dissemination of *Treponema pallidum*

**DOI:** 10.1080/22221751.2024.2382236

**Published:** 2024-07-17

**Authors:** Wei Li, Xi Luo, Xin-Qi Zheng, Qiu-Ling Li, Ze Li, Qing-Qi Meng, Yan-Li Zeng, Yu Lin, Tian-Ci Yang

**Affiliations:** a Center of Clinical Laboratory, Zhongshan Hospital Xiamen University, School of Medicine, Xiamen University, Xiamen, People’s Republic of China; b Institute of Infectious Disease, School of Medicine, Xiamen University, Xiamen, People’s Republic of China; c Xiamen Clinical Laboratory Quality Control Center, Xiamen, People’s Republic of China

**Keywords:** *Treponema pallidum*, *Treponema pallidum* protein Tp0136, angiogenesis, vascular permeability, PI3K-AKT

## Abstract

The incompletely eliminated *Treponema pallidum* (*T. pallidum*) during primary syphilis chancre infection can result in the progression of secondary, tertiary, or latent syphilis in individuals, suggesting that *T. pallidum* has successfully evaded the immune response and spread to distant sites. The mechanism underlying the dissemination of *T. pallidum* is unclear. Here, a syphilitic rabbit model dorsal-injected with recombinant Tp0136 protein or Tp0136 antibody subcutaneously was used to demonstrate the role of Tp0136 protein in promoting the dissemination of *T. pallidum* to the testis and angiogenesis *in vivo*; vascular endothelial cell line HMEC-1 was employed to display that Tp0136 protein enhances the angiogenesis. Furthermore, the three-dimensional microfluidic angiogenesis system showed that the angiogenesis would heighten vascular permeability. Then transcriptome sequencing analysis, in conjunction with cell-level validation, elucidated the critical role of the PI3K-AKT signaling pathway in the promotion of angiogenesis by Tp0136 protein, resulting in heightened permeability. These findings elucidate the strategy employed by *T. pallidum* in evading immune clearance.

## Introduction

Syphilis, a sexually transmitted disease caused by *Treponema pallidum* subspecies *pallidum* (*T. pallidum*), is a major public health concern globally [1,2]. Previous study has substantiated that *T. pallidum* can evades elimination by the host’s immune system from the initial infection sites, thereby disseminating throughout the entire organism [3], where it remains latent until an opportunity to cause relatively severe damage is presented [4]. Currently, little is known about the mechanism underlying the occurrence and development of dissemination, particularly the involvement of *T. pallidum* in the process.

Angiogenesis is essential to support channel for disseminating pathogens and tumor cells [5,6]. Excessive angiogenesis increases vascular permeability, allowing for pathogens or tumor cells to break the endothelial barrier for distal spread [7,8]. Although prior researches have demonstrated that certain membrane proteins of *T. pallidum*, like Tp47 and TpF1, facilitate angiogenesis in zebrafish models as well as cell tubular-forming models [9,10], the potential for *T. pallidum* protein-induced angiogenesis to facilitate the dissemination of *T. pallidum* and the underlying mechanisms remain to be fully understood.

*Treponema pallidum* protein Tp0136 is an adhesion protein of *T. pallidum* that plays an important role in mediating the adhesion of *T. pallidum* to endothelial cells [11], and considered as a promising syphilis vaccine candidate in inhibiting *T. pallidum* dissemination [12]. Our previous studies have demonstrated that Tp0136 actively promoted the migration of fibroblasts and microvascular endothelial cells [11,13]. The migratory potential of vascular endothelial cells plays a pivotal role in angiogenesis [14]. These findings suggest that Tp0136 protein may play a crucial role in the angiogenesis-mediated dissemination of *T. pallidum*.

In this study, we aimed to assess the effects of Tp0136 on angiogenesis and bacterial dissemination. To this end, rabbit model of syphilis was created and injected with Tp0136 recombinant proteins and antibodies. The role of Tp0136 in promoting angiogenesis and altering vascular permeability in skin lesions was elucidated, and the mechanism underlying Tp0136 activity in promoting angiogenesis and increasing vascular permeability was revealed using a microfluidic model of angiogenesis and three-dimensional cell culture. These findings will contribute to the understanding of the dissemination of *T. pallidum* and unmask the important strategy employed by *T. pallidum* to evade immune clearance in the original infection.

## Materials and methods

### Preparation of the recombinant *T. pallidum* membrane protein Tp0136

Recombinant *T. pallidum* membrane protein Tp0136 was purified and endotoxins were removed as described in a previous study [15]. Briefly, the full-length Tp0136 was directly cloned into the pEXP-5-CT-TOPO vector, then the recombined plasmid was inserted into *E. coli* BL21 strains, and grown in Luri-Bertani medium supplied with 60 μg/mL ampicillin. Protein expression was added 0.5 mmol/L isopropyl-B-Dthiogalactopyranoside (IPTG) at 25℃ for 6 h. The *E. coli* inserted with Tp0136 genes were harvested by centrifugation (6,000 g, 15 min, and 4 ℃) and lysed by a sonicator with PBS augmented with 10 μg/mL lysozyme (Solarbio, Beijing, China). The lysate was collected by centrifugation (12,000 g, 20 min, and 4 ℃). The pellet at the bottom of the tube was solubilized overnight in a buffer containing 8 mol/L urea and 0.1 mol/L Tris-HCl (pH = 8.0) at 4 ℃. Tp0136 recombinant proteins were purified from the pellet and eluted with PBS. Endotoxin contamination was removed using trace dynamic chromogenic limulus reagent (Bioendo, Xiamen, China) and was maintained at less than 0.05 endotoxin units (EUs)/mL. A bicinchoninic acid (BCA) (Takara Bio, Inc., Kusatu City, Japan) assay kit was used to estimate the concentrations.

### Anti-Tp0136 antibody directed against Tp0136 recombinant protein

The monoclonal anti-Tp0136 antibody, obtained from Boson Biotech (Xiamen, China) with a titer of 1:20,000, was utilized in this study. Prior to injection into rabbits, the antibody content in the serum of syphilis-infected rabbits was assessed as previous study discribed [16], revealing a titer of approximately 1:10,000. Consequently, the anti-Tp0136 antibody was diluted to a titer of 1:10,000 for subsequent experimental procedures.

### Establishment of the rabbit infection model

**Figure 1.**
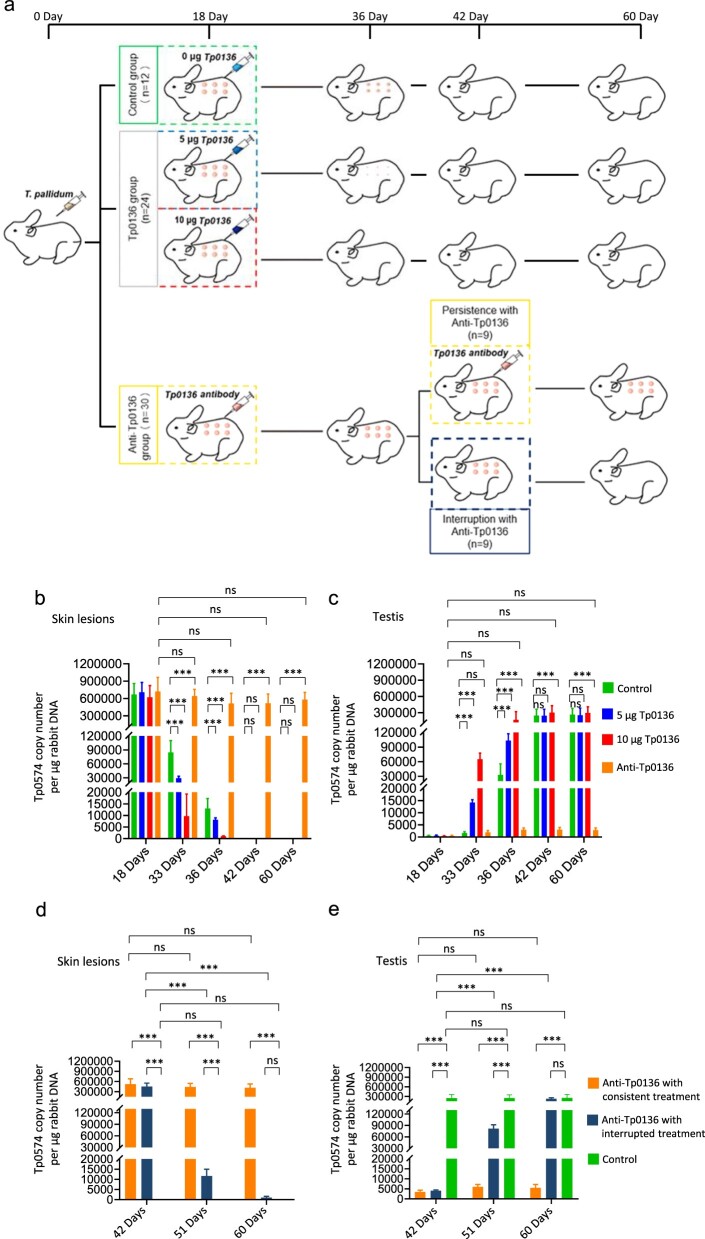
Tp0136 accelerated dissemination of *T. pallidum*. **(a)** Procedure of the rabbit infection experiment. The number of repetitions for each processing method can be found in the figure indicated by the numeric value of “n”. **(b)** Dynamic changes in the *T. pallidum* load in skin lesions on days 18, 33, 36, 42, and 60 in different groups. At each time point, three rabbits are used as biological repetitions for *T. pallidum* load detection. **(c)** Dynamic changes in the *T. pallidum* load in testes on days 18, 33, 36, 42, and 60 in different groups. At each time point, three rabbits are used as biological repetitions for *T. pallidum* load detection. **(d)** Dynamic changes in the *T. pallidum* load in skin lesions on days 42, 51, and 60 in the different anti-Tp0136 subgroups. At each time point, three rabbits are used as biological repetitions for *T. pallidum* load detection. **(e)** Dynamic changes in the *T. pallidum* load in testes on days 42, 51, and 60 in the different anti-Tp0136 subgroups. At each time point, three rabbits are used as biological repetitions for *T. pallidum* load detection. Data are expressed as the mean ± standard deviation (SD) from three independent experiments. Two-way analysis of variance (ANOVA) was used to compare the mean values of three or more groups with two independent variables. ***P < 0.001, ns; nonsignificant difference.

The *T. pallidum* Nichols strain was generously provided by Lorenzo Giacani, PhD (University of Washington, Seattle, WA, USA) and propagated in rabbits as described previously [17]. Male New Zealand rabbits (2.5 ± 0.5 kg, 13–15 weeks old, Department of Zoology, Xiamen University) with negative rapid plasma regain circle card test and *T. pallidum* particle agglutination were used for the experiments. Based on previous studies, day 18 was selected for injection of Tp0136 recombinant protein or anti-Tp0136 antibody, and to terminate the antibody action on day 42 to illustrate the role of Tp0136 protein in dissemination of *T. pallidum* [16,18–20]. The procedure used for the rabbit infection experiments is shown in Figure 1(a). Rabbit model of syphilis was created via subcutaneous inoculation of 1 × 10^6^ *T. pallidum* on rabbit backs. After 18 days of inoculation, the syphilitic rabbits were randomly divided into three groups, and were subcutaneously injected with 0 μg Tp0136 (the control group, n = 12), 5 and 10 μg Tp0136 (the Tp0136 group, n = 24), and the Tp0136 antibody (the Tp0136 antibody group, n = 30) into the skin lesion every 3 days. Three rabbits were randomly selected to be euthanized on days 18, 33, 36 and 42 of inoculation in each group, and skin lesions and testicular tissues were collected. Furthermore, the syphilitic rabbits in the Tp0136 antibody group were randomly divided into two subgroups on day 42, the Tp0136 antibody injections were interrupted in one subgroup (Anti-Tp0136 with interrupted treatment group, n = 9) and consistently administered in the other subgroup (Anti-Tp0136 with consistent treatment group, n = 9). Three syphilitic rabbits in each antibody subgroup were randomly selected to be euthanized on days 42, 51, and 60, and skin lesions and testicular tissues were collected. This study was approved by the Institutional Ethics Committee of Xiamen University, China (No. XMULAC20190129). Every effort was made to minimize the number and suffering of animals used in this study.

### Detection of the bacterial load of *T. pallidum* using real-time PCR

DNA was extracted from the skin lesions and testicular tissues for real-time PCR. Primers targeting the *Tp0574* gene of *T. pallidum* and the rabbit *matrix metalloproteinase-1* gene were used for quantitative analysis of *T. pallidum* DNA and rabbit DNA, respectively [21,22]. All the primers were purchased from Sangon Biotech (Shanghai, China). To thoroughly investigate the involvement of Tp0136 in the transmission of *T. pallidum* from lesions to the testis, a one-to-one correspondence between lesions and rabbit testis was strictly maintained in our study.

### Hematoxylin–Eosin staining

The skin tissues were fixed, dehydrated, transparent, and waxed into wax blocks, which were paraffin-sectioned to a thickness of 3**–**5 μm. The samples were stained with a hematoxylin–eosin staining kit (Solarbio, China), observed and photographed using a microscope (CX41, Olympus, Japan). In order to determine the quantity of blood vessels present in skin lesions following HE staining, we employed a methodology involving the random selection of ten samples for each treatment, observed and recorded 50 visual fields in each group. The identification of blood vessels, based on the presence of red blood cells, was determined and the average count of blood vessels under identical treatment conditions was calculated. To enhance comprehension, red arrows were utilized to denote the location of blood vessels on the HE staining images.

### Cell culture

Human microvascular endothelial cell line-1 (HMEC-1) (Procell Life Science & Technology, China) was maintained in HMEC-1 cell-specific medium (Procell Life Science & Technology, China) in a humidified incubator at 37 ℃ and 5% CO2 for 3 days, and the medium was changed every 24 h.

For the Transwell migration assay, to uphold the assay's reproducibility, a total of 8 × 10^4^ cells were seeded in the chamber for 3 days. For the three-dimensional microfluidic assay, HMEC-1 were dissociated, pelleted, and suspended in a concentration of 2 × 10^7^ cells/mL. 2 μL of the cell suspension was dispensed into the perfusion inlet and incubated for 10 days. This quantity of cells is deemed to be excessive, leading to a cell fusion rate that can achieve 100% within one day and mimic the accelerated growth of blood vessel wall cells in the following days.

To ensure the uniformity of cell fusion across experimental batches, a standardized cell inoculum (2 × 10^7^ cells/mL) was utilized, and a single pore cell from each batch was subjected to CD31 staining for cell-edge determination. The boundaries of each cell were subsequently analyzed using ImageJ software, with each cell being color-coded. The extent of cell fusion was quantified by determining the ratio of the colored area within a given visual field to the total area of that field.

### Transwell migration assay

For the experiment, HMEC-1 cells (3 × 10^4^) were seeded in 8 mm pore size Transwell chambers (Corning, USA) for 6 h [23]. The cells were fixed in 4% polymethanol solution for 15 min and stained with 0.1% crystal violet for 20 min. The number of migrating cells was observed and recorded using a microscope. The migrated cells were quantified by manual counting [24].

### Spheroid-based sprouting angiogenesis assay

Tp0136 was co-cultured with HMEC-1 cells, and the sprouting of globules was determined as described in a previous study [23]. Briefly, HMEC-1 cells were added to the fresh conical tube and treated with or without 10 μg/mL recombinant protein Tp0136 then incubated in a humidified cell culture incubator set at 37℃ and 5% CO_2_ for 24 h. Spheroids were embedded in the 24-well plate, cultured with collagen medium containing 20% FBS in a humidified cell culture, and incubated at 37℃ for 24 h. The samples were fixed with paraformaldehyde and the budding microspheres were observed under a microscope. Photographs were used to quantify the total sprout length with ImageJ by measuring the cumulative sprout length of all sprouts per spheroid. The cumulative length of sprouts was measured and the mean value was calculated to assess the disparity between the group treated with Tp0136 recombinant protein and the untreated group.

### Tube formation assay

A total of 20,000 HMEC-1 cells were pre-incubated with 200 μL of MCBD13 endothelial basal medium (ScienCell) supplied with 10% fetal bovine serum (FBS), 2 mM L-glutamine, and 1 μg/mL hydrocortisone, suspensions were inoculated into 96-well plates pre-coated with 50 µL of growth factor-reduced Matrigel (Corning, USA) for 6 h and stained with calcein-AM (MedChemExpress, USA) [9]. The formation of capillary-like structures was observed and imaged using a fluorescence microscope.

To quantify the tubular networks, ImageJ software was utilized. The process involved applying flat-field correction, initiating with Gaussian blurring, and utilizing edge detection to generate a binary image of tubules. Subsequently, dilated skeletonization of tubules was performed to streamline the network quantification. The final step involved quantifying the nodes by assessing the number of branch points (3-, 4-, or 5-way) and incorporating loop counts.

### Three-dimensional microfluidic angiogenesis system and detection of vascular permeability

As previously described, angiogenesis analysis was based on a microfluidic model and three-dimensional cell culture to simulate angiogenesis *in vivo* [9,25]. HMEC-1 were dissociated, pelleted, and suspended in a concentration of 2 × 10^7^ cells/mL. 2 μL of the cell suspension was dispensed into the perfusion inlet and incubated for 10 days. When the cells formed a stable monolayer, stimulation factors were added to the bottom perfusion channel, which was then placed in a shaker for continuous perfusion. On day 10 after stimulation, 50 mL of 150 kDa TRITC-Dextran solution (Sigma-Aldrich, USA) was added to the perfusion channel inlet and time-lapse images were acquired at 30-second intervals using a confocal microscope. The cytoskeleton and nuclei were stained via immunofluorescence, using a Phalloidin-iFluor 555 reagent (Abcam, UK) and an anti-fluorescence quenching blocking solution containing DAPI (Beyotime, China), respectively. A laser confocal scanning microscope (LSM780, Zeiss, Germany) was used to obtain three-dimensional scanning images.

### Detection of monolayer endothelial barrier permeability

To determine the endothelial barrier permeability, HMEC-1 cell suspension was inoculated in the upper chamber of the 0.4 mm pore size Transwell chamber (Corning, USA). Different concentrations of the recombinant protein Tp0136 (2.5, 5, and 10 mg/mL) and PBS were added to the upper chamber and cultured for 6 h. Moreover, 10 mg/mL Tp0136 was added to the upper chamber to co-incubate with HMEC-1 cells for different durations (0, 1, 2, 3, 4, 5, and 6 h). Then 100 mL Evans Blue-bovine serum albumin dye was added to the upper chamber, and all liquids in the lower chamber were collected after 30 min. The absorbance was measured at 650 nm.

### RNA-sequencing data analysis and visualization

RNA sequencing data were analyzed and visualized based on the methodology reported in a previous study [26]. Paired-end sequencing reads were trimmed, and quality was checked using Fastqc (version 0.11.7). Alignment to the human genome was performed using STAR software (version 2.5.2a). Differential gene expression analysis was performed using RStudio software (version 1.0.153). For the study of differentially expressed genes exposed to the Tp0136 recombinant protein, we conducted a comparative analysis between the group treated with the Tp0136 recombinant protein and the untreated group, identifying a gene subset with a significance level of *p* < 0.001. To identify genes of greater research significance and more pronounced changes, those with a *p *< 0.001 and a gene fold-change ratio ≥1.5 following treatment with Tp0136 recombinant protein were selected as the target gene set. Subsequent sequencing based on the change ratio resulted in the selection of the top 100 genes for further analysis using Gene Ontology (GO) and Kyoto Encyclopedia of Genes and Genomes (KEGG) pathways. The GO and enrichment analyses of RNA sequencing data were conducted using Panther (http://pantherdb.org/) and Enrichr (https://amp.pharm.mssm.edu/Enrichr/) on specified gene lists. To more clearly observe the enriched function term of differentially expressed genes, we filtered the function term with a significance level of *p *< 0.001 in the GO enrichment analysis. In conducting differentially expressed gene pathway enrichment analysis, we employed a methodology similar to that utilized in GO analysis to identify the genes exhibiting the most significant changes. Subsequently, these identified genes were subjected to analysis using the KEGG database. The raw sequence data reported in this paper have been deposited in the Genome Sequence Archive (Genomics, Proteomics & Bioinformatics 2021) in National Genomics Data Center (Nucleic Acids Res 2022), China National Center for Bioinformation / Beijing Institute of Genomics, Chinese Academy of Sciences (GSA-Human: HRA005158) that are publicly accessible at https://ngdc.cncb.ac.cn/gsa-human.

### Western blotting

Western blotting was performed as previously described [27]. PI3K, P-PI3K, AKT, p-AKT (Ser473), p-AKT (Thr308), and GAPDH antibodies were purchased from Cell Signaling Technology. Bands were visualized using the Gel Recording System (Bio-Rad, USA) or iBright™ CL750 Imaging System (Thermo Fisher Scientific, USA).

### Statistical analysis

All experiments were performed at least three times, and the data were presented as mean ± standard deviation. GraphPad Prism 8.0 (GraphPad Software, La Jolla, CA, USA) was used for all the statistical analyses. Student's *t* test was used when two groups were compared. One-way analysis of variance (ANOVA) was used to compare the mean values of three or more groups with one independent variable. Two-way ANOVA was used to compare the mean values three or more groups with two independent variables. Post hoc comparisons were performed using Tukey's test. Statistical significance was defined as a two-sided *P* value of < 0.05.

## Data availability

The RNA-seq sequencing data reported in this paper have been deposited in the Genome Sequence Archive (Genomics, Proteomics & Bioinformatics 2021) in National Genomics Data Center (Nucleic Acids Res 2022), China National Center for Bioinformation / Beijing Institute of Genomics, Chinese Academy of Sciences (GSA-Human: HRA005158) that are publicly accessible at https://ngdc.cncb.ac.cn/gsa-human. Source data are provided with this paper. The raw immunofluorescence and statistical figures can be obtained on the figshare website, DOI:10.6084/m9.figshare.26197763.

## Results

### Tp0136 accelerated the dissemination of *T. pallidum*

In our previous study, it was observed that after inoculation with the Nichols strain, lesions in rabbit models reached maximum ulcers after about 18 days, on which it was considered that *T. pallidum* was clustered in the ulcers, and fully healed around 42 days after inoculation with *T. pallidum*, on which it was considered that *T. pallidum* was completely removed from the ulcer [16,18,19]. Furthermore, Ke *et al*. reported in their study that the most elevated concentrations of *T. pallidum* were detected in lesions approximately 15-18 days post-inoculation [20]. To explore the role of Tp0136 in the dissemination of *T. pallidum*, rabbit model of syphilis with dorsal subcutaneous inoculation for 18 days of inoculation with *T. pallidum*. Subsequently, either Tp0136 recombinant protein or Tp0136 antibody was injected subcutaneously into the lesions every 3 days. The *T. pallidum* load was measured from the skin lesions at the inoculation sites on days 18, 33, 36, 42, and 60 in different groups. On day 18, the skin lesions of the syphilitic rabbits exhibited high levels of *T. pallidum* load (Figure 1(b)). The *T. pallidum* load on the skin lesions decreased significantly on days 33 and 36 in the Tp0136 group (*P *< 0.001) (Figure 1(b)). With an increase in the inoculated Tp0136 concentration, the *T. pallidum* load decreased significantly (*P *< 0.001), whereas the *T. pallidum* load remained high on days 33, 36, 42 and 60 in the antibody group, with no significant difference compared to that on day 18 (*P* > 0.05) (Figure 1(b)). The decreasing *T. pallidum* load in the skin lesions and last until day 42, the time-point on which the *T. pallidum* was undetectable even in the Control group, indicating that day 42 marks the end of Tp0136 protein’s function in promoting the dissemination of *T. pallidum* from the skin lesions. To track the dissemination of *T. pallidum*, the *T. pallidum* load in the testes was evaluated on days 18, 33, 36, 42 and 60. The *T. pallidum* load in the testes showed an opposite trend to that in skin lesions, and the *T. pallidum* load was barely measurable in the testicular tissue on day 18. The *T. pallidum* load in the testes treated with Tp0136 recombinant protein was significantly increased at days 33, 36, 42 and 60 compared to day 18 and increased with increasing concentrations of Tp0136 treatment (*P* < 0.001). In the antibody group, the *T. pallidum* load in the testes on days 33, 36, 42 and 60 was not significantly different from that on day 18 (*P* > 0.05), and the *T. pallidum* load was barely measurable (Figure 1(c)). After the discontinuation of the Tp0136 antibody injection on day 42, the *T. pallidum* load was significantly reduced on days 51 and 60 in the skin lesions (*P* < 0.001), and there was no significant difference in the *T. pallidum* load within the lesion compare to those of the Control groups on day 60 (Figure 1(d)); however, the *T. pallidum* load of the skin lesions remained high in the subgroup treated consistently with Tp0136 antibodies, and there was no significant difference at different time points (*P *> 0.05) (Figure 1(d)). The *T. pallidum* load in the testes increased significantly on days 51 and 60 after discontinuation of the Tp0136 antibody injection (*P* < 0.001), with the testicular *T. pallidum* load reaching levels comparable to those of the Control group on day 60, whereas the *T. pallidum* load of the testes remained relatively low in the treatment subgroup with consistent anti-Tp0136, and no significant difference was observed at different time points (Figure 1(e)). These results indicate that Tp0136 accelerates the dissemination of *T. pallidum* in the infected rabbits.

### Tp0136 promoted angiogenesis *in vivo* and *in vitro*

**Figure 2.**
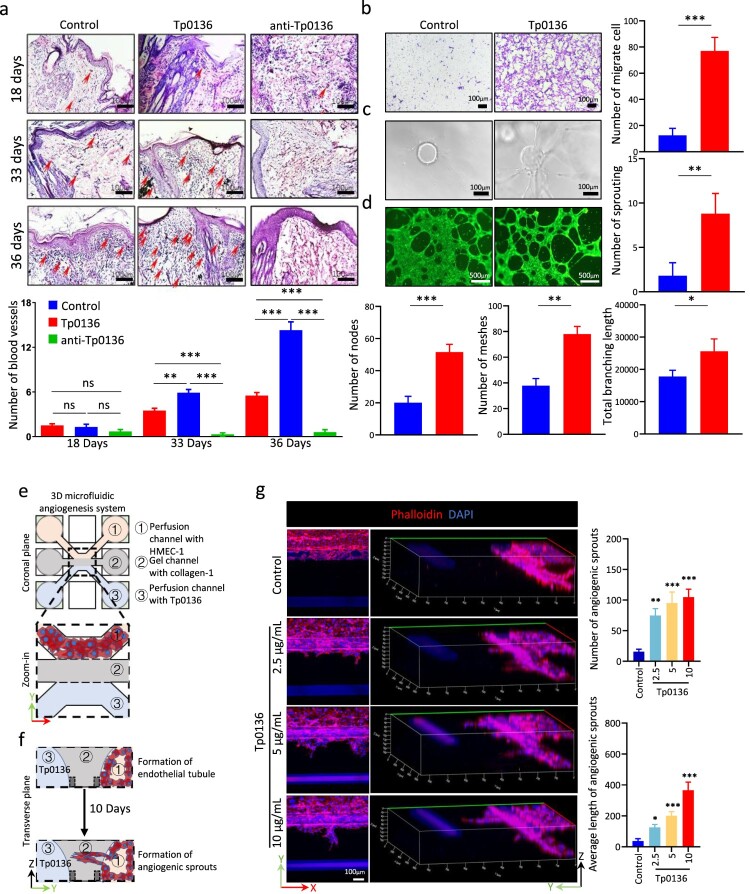
Tp0136 promoted angiogenesis *in vivo* and *in vitro*. **(a)** Effect of Tp0136 protein and antibody on blood vessels in lesions. Red arrows indicate the blood vessels. The bar graph indicates the number of the blood vessels. Scale bars = 100 mm. **(b)** Effect of Tp0136 on cell migration using transwell migration assay. The bar graph indicates the crystal violet area. Scale bars = 100 mm. The experiment was repeated three times independently for each group. **(c)** Effect of Tp0136 on spheroid-based sprouting. The bar graph indicates the number of sprouting. Scale bars = 100 mm. The experiment was repeated three times independently for each group. **(d)** Effect of Tp0136 on tubule formation. Tp0136 (10 mg/mL) was coincubated with HMEC-1 cells for 6 h, after which tubule formation was stained using Calcein AM. Bar graphs indicate the number of nodes, the number of meshes, and the total branching length. Scale bars = 500 mm. The experiment was repeated three times independently for each group. **(e-f)** Three-dimensional microfluidic angiogenesis system of angiogenesis processed by Tp0136. **(g)** Effect of Tp0136 on three-dimensional angiogenesis. The bar graph indicates the number and the average length of angiogenic sprouts. Phalloidin-iFluor 555-red, DAPI-blue. Scale bars = 100 mm. The experiment was repeated three times independently for each group. Data are expressed as the mean ± SD from three independent experiments. Student's *t* test was used to compare data between two groups. One-way ANOVA was used to compare the mean values of three or more groups with one independent variable. *P vs. Control, *, *P*<0.05, **, *P*<0.01, ***, *P*<0.001.

Cutaneous syphilitic lesions were observed to be highly angiogenic [28] and the dissemination of pathogens closely associated with excessive angiogenesis [29,30]. In the animal experiments described above, Hematoxylin–Eosin staining was involved in the observation of blood vessels in skin lesions, with the determination of their location based on the presence or absence of red blood cells within the tubular structure. It revealed that the number of blood vessels in the 10 μg Tp0136 recombinant protein group was significantly higher than that in the control group on days 33 and 36, the quantity of blood vessels in the group treated with the anti-TP0136 antibody consistently remained at a low level (Figure 2(a), the red arrows represent the locations of blood vessels). The pro-angiogenic ability of Tp0136 recombinant protein *in vitro* was further evaluated using transwell migration, spheroid-based sprouting angiogenesis, and tube formation assays. The results of the transwell migration assay revealed that the number of cells migrated across the porous membrane increased significantly after treatment with 10 μg/mL Tp0136 recombinant protein (*P* < 0.001) (Figure 2(b)). The endothelial cell budding experiment exhibited an increased number of sprouting around the beads after 10 μg/mL Tp0136 recombinant protein treatment (*P* < 0.01) (Figure 2(c)). In addition, the tube formation assay revealed a significant increase in the number of nodes (*P* < 0.01), meshes (*P* < 0.05), and branch lengths (*P* < 0.01) of the network structure formed after Tp0136 recombinant protein treatment (Figure 2(d)). Furthermore, a three-dimensional microfluidic angiogenesis system was used to reproduce all the angiogenic events induced by Tp0136 recombinant protein (Figure 2(e,f)). Vascular structures, including tip cell formation, directed germination, and lumen formation, were formed by co-culturing Tp0136 recombinant protein with endothelial cells for 10 days. The number and average length of angiogenic sprouts with prominent filamentous pseudopods increased significantly with increasing Tp0136 recombinant protein concentration (*P* < 0.05–0.001) (Figure 2(g)). These results indicate that Tp0136 contributes to promoting angiogenesis *in vivo* and *in vitro*.

### Tp0136 increased vascular permeability with angiogenesis

**Figure 3.**
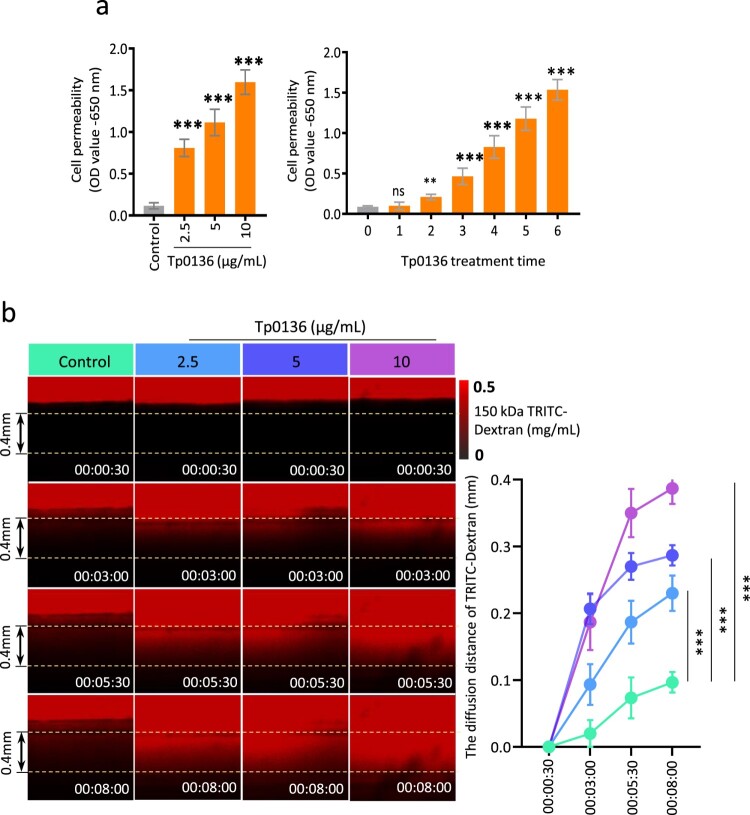
**Tp0136 increased vascular permeability with angiogenesis. (a)** Effect of Tp0136 on vascular permeability detected using Evans blue-bovine serum albumin. Left: Vascular permeability in HMEC-1 cells stimulated with different concentrations of Tp0136 for 6 h. Right: Vascular permeability in HMEC-1 cells incubated with 10 mg/mL Tp0136 for different durations. The experiment was repeated three times independently for each group. **(b)** Effect of Tp0136 on vascular permeability detected using three-dimensional angiogenesis analysis. The pseudo-colored fluorescent images after 0 and 8 min after the addition of the dextran solutions. Time is indicated in h, min, and s. Data are expressed as the mean ± SD from three independent experiments. One-way ANOVA was used to compare mean values of three or more groups with one independent variable. * *P* < 0.05, ** *P* < 0.01, *** *P* < 0.001, ns; nonsignificant difference.

Angiogenesis disrupts the integrity of the pre-existing vessels, leading to increased vascular permeability, which facilitates the spread of pathogens [31]. To examine the effect of Tp0136 on vascular permeability, an endothelial barrier model composed of HMEC-1 cells was constructed *in vitro*, where different concentrations of Tp0136 recombinant protein were added and co-incubated with endothelial cells. The permeability of the endothelial barrier was detected using Evans blue bovine serum albumin. As shown in Figure 3(a), the permeability of the monolayer endothelial barrier increased significantly with increasing Tp0136 recombinant protein concentration. The permeability of the endothelial barrier significantly increased when the stimulation concentration of Tp0136 recombinant protein was 2.5 μg/mL (*P* < 0.001) and peaked when the stimulation concentration of Tp0136 recombinant protein was 10 μg/mL (*P* < 0.001). Upon further investigation, Tp0136 recombinant protein was found to significantly increase endothelial barrier permeability in a time-dependent manner. The permeability of the endothelial barrier significantly increased when the stimulation time of 10 μg/mL Tp0136 recombinant protein was 3 h (*P* < 0.01) and peaked when the stimulation time of 10 μg/mL Tp0136 recombinant protein was 6 h (*P* < 0.001). Further, Tp0136 recombinant protein and endothelial cells were cocultured in a three-dimensional microfluidic angiogenesis system to form vascular-like structures. Thereafter, 0.5 mg/mL 150 kDa TRITC-dextran solution was perfused into the main vessels to monitor the effect of Tp0136 on vascular integrity after promoting angiogenesis. When the nascent buds are not tightly attached to the main vessels, resulting in increased vascular permeability, TRITC-dextran exudation is observed in the collagen and increases over time, resulting in an increased rate of exudation in neovascularization formed by Tp0136 recombinant protein treatment (Figure 3(b)). This suggests that Tp0136 drives angiogenesis in HMEC-1 cells, leading to a significant increase in endothelial barrier permeability. These results demonstrate that Tp0136 increases vascular permeability via angiogenesis.

### Tp0136 promoted angiogenesis via PI3K-AKT leading to an increase in vascular permeability

**Figure 4.**
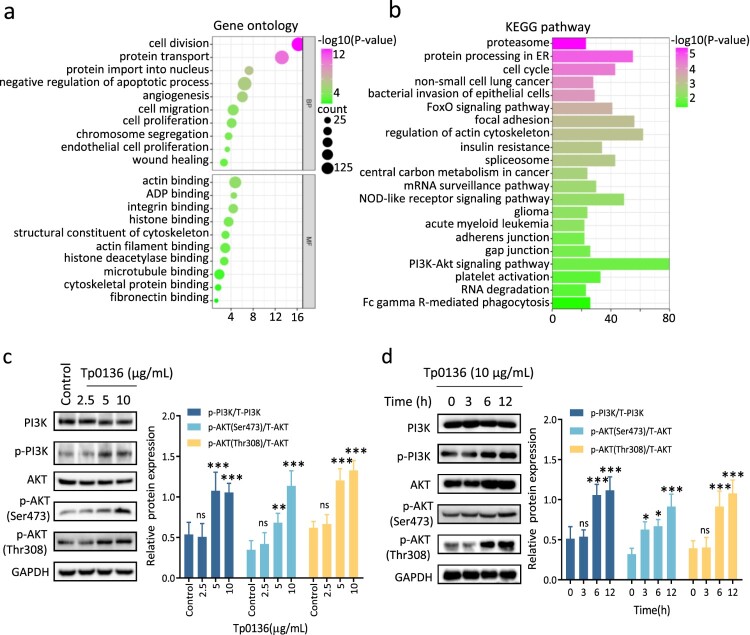
**Expression of PI3K/AKT signaling pathway in Tp0136-induced angiogenesis. (a)** GO analysis of the first 3000 differentially expressed genes after Tp0136 treatment in HMEC-1 cells. **(b)** KEGG pathway analysis of the first 3000 differentially expressed genes after Tp0136 treatment in HMEC-1 cells. **(c)** Expression of the PI3K, p-PI3K, AKT, and p-AKT proteins in HMEC-1 cells stimulated with different concentrations of Tp0136 revealed using western blotting. The bar graphs on the right indicate the ratios of the phosphorylated proteins versus the total proteins. The experiment was repeated three times independently for each group. **(d)** Expression of the PI3K, p-PI3K, AKT, and p-AKT proteins in HMEC-1 cells stimulated with Tp0136 for different durations using western blotting. The experiment was repeated three times independently for each group. Data are expressed as the mean ± SD from three independent experiments. Two-way ANOVA was used to compare the mean values of three or more groups with two independent variables. *P vs. Control, * *P* < 0.05, ** *P* < 0.01, *** *P* < 0.001, ns; nonsignificant difference.

To reveal the molecular mechanism through which Tp0136 promotes angiogenesis in HMEC-1 cells, genes with differential expression were screened using RNA sequencing. The first 3000 differentially expressed genes were subjected to GO analysis, which showed that the differentially expressed genes were mainly enriched in cell division, protein transport, protein import into the nucleus, negative regulation of apoptotic processes, and angiogenesis (Figure 4(a)). In addition, the first 3000 differentially expressed genes were subjected to KEGG analysis. As shown in Figure 4(b), the genes were mainly enriched in the PI3K/AKT signaling pathway, regulation of the actin cytoskeleton, focal adhesion, protein processing in the endoplasmic reticulum, NOD-like receptor signaling pathway, cell cycle, and FoxO signaling pathway. Studies have revealed that the PI3K/Akt signaling pathway is closely related to angiogenesis [32]. Our study showed that Tp0136 recombinant protein increased the expression of p-PI3K and p-AKT in HMEC-1 cells in a dose-dependent manner. The protein expression of p-PI3K was significantly increased at 5 μg/mL Tp0136 (*P* < 0.001) and peaked at 10 μg/mL Tp0136 (*P* < 0.001). The protein expression of p-AKT (Ser473) and p-AKT (Thr308) increased significantly at 5 μg/mL Tp0136 recombinant protein (*P* < 0.01) and peaked at 10 μg/mL Tp0136 recombinant protein (*P* < 0.001) (Figure 4(c)). Moreover, Tp0136 recombinant protein increased the expression of p-PI3K and p-AKT in a time-dependent manner. The protein expression levels of p-PI3K and p-AKT (Thr308) were significantly increased at 6 h (*P* < 0.001) and peaked at 12 h (*P* < 0.001). The protein expression of p-AKT (Ser473) significantly increased at 3 h (*P* < 0.05) and peaked at 12 h (*P* < 0.001) (Figure 4(d)).

**Figure 5.**
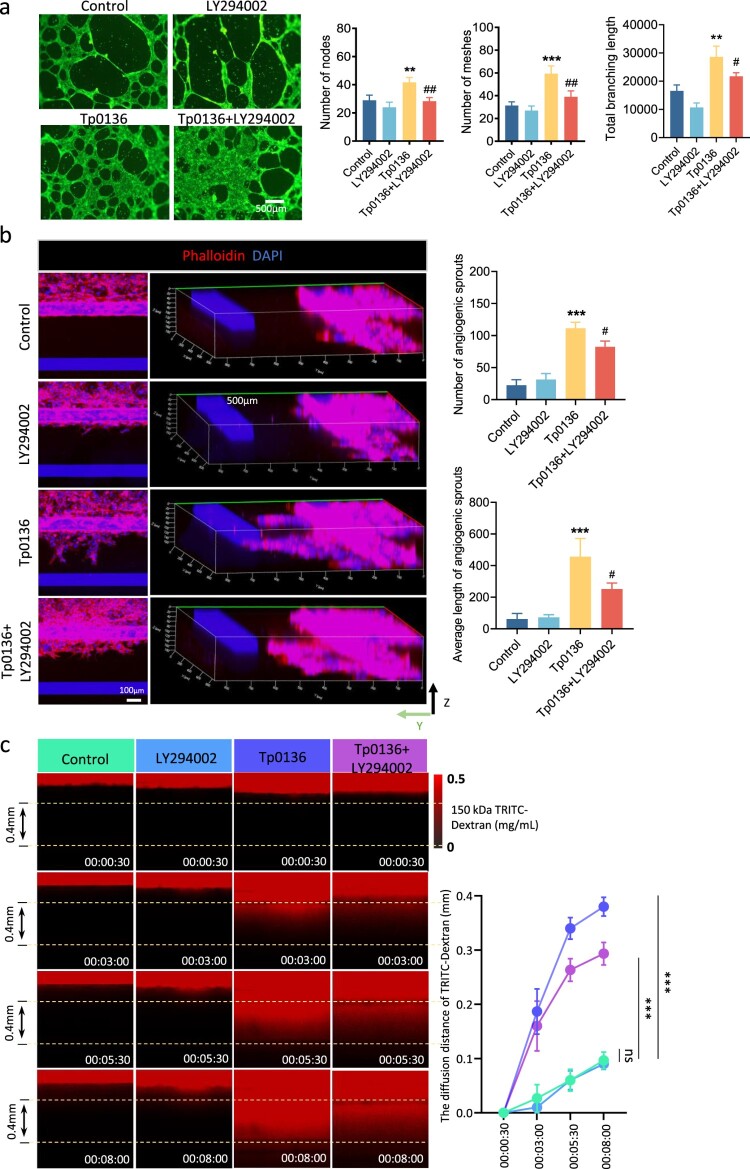
**Tp0136 promoted angiogenesis via PI3K/Akt leading to the increase in vascular permeability. (a)** Effect of LY294002 on Tp0136-induced tubule formation. Scale bars = 500 mm. Bar graphs indicate the number of nodes, the number of meshes, and the total branching length in tubule formation. The experiment was repeated three times independently for each group. **(b)** Effect of LY294002 on Tp0136-induced angiogenesis was analyzed using three-dimensional angiogenesis analysis. The bar graph shows the number and the average length of angiogenic sprouts. Phalloidin-iFluor 555-red, DAPI-blue. Scale bars = 100 mm. The experiment was repeated three times independently for each group. **(c)** Effect of LY294002 on Tp0136-induced vascular permeability in three-dimensional microfluidic angiogenesis system. Time is indicated in h, min, and s. Data are expressed as the mean ± SD from three independent experiments. One-way ANOVA was used to compare the mean values of three or more groups with one independent variable. * *P* vs. Control, ** *P* < 0.01, *** *P* < 0.001. # *P* vs. Tp0136, # *P* < 0.05, ## *P* < 0.01.

To investigate whether the PI3K-AKT signaling pathway is involved in the regulation of angiogenesis by Tp0136, HMEC-1 cells were treated with PI3K-AKT inhibitor LY294002 and then cocultured with Tp0136 recombinant protein, and the formation of tubular structures was detected using a tube formation assay. Compared to the Tp0136 group, the number of nodes (*P* < 0.01), meshes (*P* < 0.01), and branch lengths (*P *< 0.05) of the network structure on the matrix glue were significantly reduced after treatment with PI3K-AKT inhibitor LY294002 (Figure 5(a)). In addition, the results of the three-dimensional microfluidic angiogenesis system demonstrated that treatment with the PI3K-AKT inhibitor LY294002 resulted in a significant reduction in the number and average length of angiogenic shoots induced by Tp0136 recombinant protein (*P* < 0.05) (Figure 5(b)). TRITC-dextran solution was perfused into the main vessels to further assess vascular permeability. The results showed a significantly low rate of TRITC-dextran osmosis in the neovascularization formed after treatment with PI3K-AKT inhibitor LY294002 compared with the Tp0136 group (Figure 5(c)). These data suggest that Tp0136 promotes angiogenesis via the PI3K/AKT signaling pathway, leading to increased vascular permeability.

## Discussion

Angiogenesis occurring at the initial site of infection by pathogenic microorganisms serve as an indicator of pathogenic microorganism dissemination [33]. Previous scholarly research has indicated that the presence of angiogenesis in secondary syphilis lesions coincides with the process of lesion healing [28], and considered that local inflammation prompts the angiogenesis [34]. However, it is noteworthy that patients continue to exhibit secondary and tertiary syphilis, accompanied by secondary systemic infection, thereby implying that angiogenesis in skin lesions may facilitate the dissemination of *T. pallidum* [35]. In this study, Tp0136 protein and antibodies were infused subcutaneously into lesions every 3 days for 18 days after *T. pallidum* inoculation (when lesion ulceration reached its maximum) in New Zealand rabbits. The assessment of the intrinsic *T. pallidum* burden of the lesion demonstrated that the administration of Tp0136 led to a progressive and accelerated reduction in *T. pallidum* load within the lesion. Simultaneously, the *T. pallidum* load in the distal organ testes gradually increased, indicating the dissemination of *T. pallidum* from the original inoculum site to the distal tissues. This suggests that Tp0136 plays a role in accelerating this phenomenon. Remarkably, skin lesions that underwent continuous injection of the Tp0136 antibody exhibited a persistent *T. pallidum* load on the affected areas. However, no significant changes in the *T. pallidum* load were observed in the distal organ testes. Upon the discontinuation of Tp0136 antibody injection on day 42, a progressive migration of *T. pallidum* from the cutaneous lesions to the remote organ was observed throughout this timeframe. Previous studies have reported that *T. pallidum* protein Tp0136 promoted fibroblast migration [9,13]. Inflammatory skin changes recruit activated immune cells to the infected area to destroy *T. pallidum*. Actively triggering angiogenesis may be a strategy by which *T. pallidum* avoids immune cell hunting through active escape, which may be a beneficial option for *T. pallidum*, both to help it spread and escape the immune system.

Angiogenesis is reported as a supporting channel with enlarged permeability for the spread of pathogens and tumor cells [36,37]. Angiogenesis disrupts the integrity of existing blood vessels, leading to increased vascular permeability and favoring the spread of pathogens or tumor cells [6,8,38]. In the process of *T. pallidum* breaking the endothelial barrier, the microvascular endothelial barrier may be in a state of hyperpermeability. Fitzgerald confirmed that *T. pallidum* promoted the occurrence of vascular leakage [39]. In this study, the number of blood vessels in syphilitic rabbits injected with Tp0136 was significantly higher on days 33 and 36, suggesting that Tp0136 promotes angiogenesis *in vivo*. The angiogenic process of Tp0136 *in vitro* was simulated using a three-dimensional microfluidic angiogenesis system, and Tp0136 was found to promote angiogenesis, leading to increased vascular permeability. Triggering angiogenesis at the onset of infection may be a strategy by which a pathogen evades immune clearance at the primary infection site and spreads distally, thereby achieving occult infection. By initiating angiogenesis, the pathogen not only increases the delivery of nutrients favorable for its growth but also establishes conduits that allow it to spread to distant tissues, leading to the establishment of multiple foci of infection. This mechanism has been elucidated in the pathogenesis of *Mycobacterium tuberculosis* [40,41].

Recent studies have shown that the PI3K/AKT signaling pathway plays an important role in angiogenesis [42]. PI3K is activated and contributes to the production of the second messenger, PIP3, which in turn activates AKT. Activated AKT regulates vascular endothelial cell proliferation, migration, and survival and promotes new vasculature formation through multiple signaling pathways [43]. Pathogenic microorganisms play a significant role in activating the PI3K/AKT signaling pathway, which in turn promotes angiogenesis for self-propagation and dissemination. For instance, *Mycobacterium* demonstrates the ability to reproduce and spread through this mechanism [44,45]. Additionally, previous research has shown that *T. pallidum* can also activate the PI3K/AKT signaling pathway to stimulate angiogenesis [46]. This study analyzed the genome-wide information of Tp0136-regulated HMEC-1 cells using RNA sequencing and revealed that differentially expressed genes were highly enriched in the PI3K/AKT signaling pathway. In addition, Tp0136 promoted the expression of p-PI3K and p-AKT. Further studies demonstrated that an inhibition of the PI3K/AKT signaling pathway inhibited Tp0136-induced angiogenesis and increased vascular permeability. These findings suggest that Tp0136 promoted angiogenesis through PI3K-AKT leading to increased vascular permeability. Notably, aberrant angiogenesis resulted in an increase in vascular permeability caused by overactivation of the PI3K/AKT signaling pathway [47]. Overactivation of the PI3K-AKT signaling pathway causes dysregulation of adhesion molecule expression in vascular endothelial cells, leading to increased permeability of the vessel wall [48].

To our knowledge, this study is the first to demonstrate that *T. pallidum* protein Tp0136 promotes the dissemination of *T. pallidum* through angiogenesis and increased vascular permeability. However, this study has several limitations. The ability of Tp0136 to promote angiogenesis was observed both *in vivo* and *in vitro*, and increased vascular permeability resulting from Tp0136-promoted angiogenesis was verified *in vitro* using a three-dimensional microfluidic angiogenesis system. Although numerous studies have found angiogenesis in pathogen dissemination to be beneficial, further research is required to comprehensively elucidate the specific mechanisms by which *T. pallidum* spreads to distant tissues with the aid of high permeability during angiogenesis. Furthermore, the distal spread of *T. pallidum*, which is considered as an important manifestation of evade immune clearance. Although *T. pallidum* may perform this strategy through the Tp0136 protein, the potential impact of a Tp0136 gene knockout strain on the dissemination of *T. pallidum* can be elucidated through the observation of interactions between the wild-type strain and the knockout strain. This investigation may provide insights into the role of Tp0136 in promoting dissemination. However, the current limitations in culturing *T. pallidum in vitro* for extended periods hinder the advancement of gene knockout technology targeting Tp0136. As a result, this study only focused on analyzing the impact of recombinant protein Tp0136 and anti-Tp0136 antibody on treponemal dissemination, confirming the role of Tp0136 protein in promoting dissemination. Finally, owing to the limitations of the rabbit model for syphilis infection, the present study lacked corresponding experiments *in vivo* to elucidate the detailed mechanism by which Tp0136 promotes angiogenesis, leading to increased vascular permeability in the pathogenesis of syphilis. Zebrafish is commonly employed in research on angiogenesis. In our prior study, we utilized the zebrafish model to confirm the angiogenic properties of a different protein from *T. pallidum* [9,49]. In this study, a rabbit model was employed to examine the role of Tp0136 in promoting angiogenesis by *T. pallidum*, facilitating the dissemination of the pathogen to distal organs. Hence, for distinct research objectives, rabbit models were exclusively selected in this study. Additionally, the finding in the 3D angiogenesis experimental model showed Tp0136 promoted the angiogenesis to facilitate the permeability which aligned with the outcomes observed in rabbit models. It is important to mention that a previous study conducted by our research team demonstrated that *T. pallidum* protein Tp47 was involved in angiogenesis via the ROS pathway [9]. While this study did not provide evidence regarding whether Tp47 promotes the dissemination of *T. pallidum* through angiogenesis, it is plausible that Tp47 shares similar functional characteristics with Tp0136, as the pathogenic mechanism of *T. pallidum* likely involves a multifaceted interplay of various proteins.

**Figure 6.**
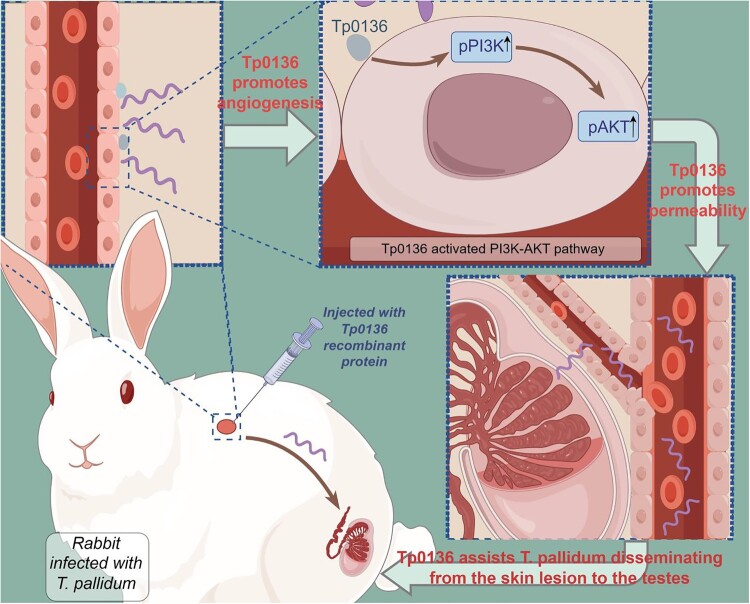
***Treponema pallidum* protein Tp0136 actively promotes the dissemination of *T. pallidum* by increasing vascular permeability.** Tp0136 accelerated the dissemination of *T. pallidum*, and Tp0136 facilitated angiogenesis via PI3K-AKT leading to increased vascular permeability favoring the dissemination of *T. pallidum*.

In summary, the present study revealed that Tp0136 accelerated the dissemination of *T. pallidum*. The results show that Tp0136 facilitated angiogenesis via PI3K-AKT, leading to increased vascular permeability and favoring the dissemination of *T. pallidum* (Figure 6). These findings may elucidate the distal dissemination of *T. pallidum* that evades clearance by the immune system during syphilis and unmasks the important strategy of *T. pallidum* to escape immune clearance in the original infection. Consequently, targeting the Tp0136 protein or its associated angiogenic signaling pathways may represent a novel strategy for the prevention and treatment of syphilis. Inhibiting the function of Tp0136 or obstructing its associated signaling pathways, like PI3K-AKT, could potentially mitigate the dissemination of *T. pallidum*, thereby offering novel insights for the treatment and prevention of syphilis. Future advancements in the development of specific inhibitors or pharmacological agents targeting Tp0136 or its signaling pathways may yield significant breakthroughs in the prevention and control of syphilis.

## Conclusion

Syphilis has a highly insidious nature, and the dissemination of *T. pallidum* between the infected sites and distal organs is an important factor in the progression of syphilis. The objective of this study was to elucidate the role of Tp0136 in expediting the promoting the dissemination of *T. pallidum*, and facilitating angiogenesis both *in vivo* and *in vitro*. Additionally, it was observed that angiogenesis resulted in heightened vascular permeability, with the activation of PI3K/AKT signaling pathway playing a role in regulating angiogenesis and enhancing vascular permeability. This investigation provides novel insights into the immune evasion strategies employed by *T. pallidum* during infection.

## Abbreviations

PI3K: phosphatidylinositol-4,5-bisphosphate 3-kinase; Akt: serine/threonine protein kinase B; HMEC-1: human microvascular endothelial cell line-1; TRITC: Tetramethylrhodamine; RT–PCR: real-time polymerase chain reaction.
